# Quantifying microglial morphology: an insight into function

**DOI:** 10.1093/cei/uxae023

**Published:** 2024-03-08

**Authors:** Tabitha R F Green, Rachel K Rowe

**Affiliations:** Department of Integrative Physiology, The University of Colorado Boulder, Boulder, CO, USA; Department of Integrative Physiology, The University of Colorado Boulder, Boulder, CO, USA

**Keywords:** immunohistochemistry, glia, morphology, histology

## Abstract

Microglia are specialized immune cells unique to the central nervous system (CNS). Microglia have a highly plastic morphology that changes rapidly in response to injury or infection. Qualitative and quantitative measurements of ever-changing microglial morphology are considered a cornerstone of many microglia-centric research studies. The distinctive morphological variations seen in microglia are a useful marker of inflammation and severity of tissue damage. Although a wide array of damage-associated microglial morphologies has been documented, the exact functions of these distinct morphologies are not fully understood. In this review, we discuss how microglia morphology is not synonymous with microglia function, however, morphological outcomes can be used to make inferences about microglial function. For a comprehensive examination of the reactive status of a microglial cell, both histological and genetic approaches should be combined. However, the importance of quality immunohistochemistry-based analyses should not be overlooked as they can succinctly answer many research questions.

## Introduction

**Figure 1.**
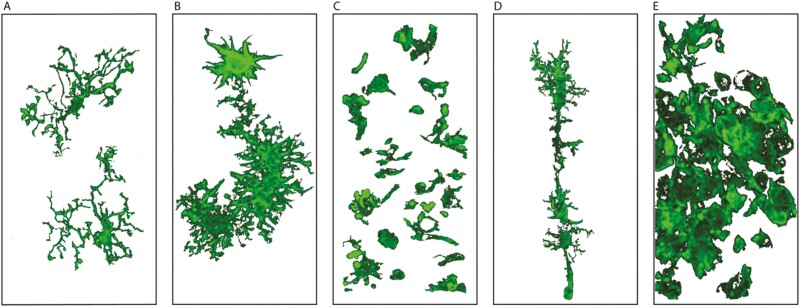
Microglial morphologies. Microglial morphologies adapted from photomicrographs of Iba1-stained cells. (**A**) Ramified microglia. (**B**) Bushy or hypertrophic microglia. (**C**) Ameboid microglia. (**D**) Rod microglia. (**E**) Microglial scar. 3D projected images were processed in ZEN blue and edited in Illustrator. These images should be treated as representative diagrams.

Microglia are phagocytic immune cells unique to the central nervous system (CNS) [1]. Microglia actively survey the microenvironment of the brain to detect damage or pathogen-associated signals [2–4]. Upon detecting perturbations in the microenvironment, microglia quickly respond to protect the brain from further tissue damage and restore neuronal homeostasis [5, 6]. Rio-Hortega discovered microglia in 1919, then he developed the silver carbonate staining method that allowed detailed visualization of microglial morphology [7]. Since this discovery, immunohistochemistry has become one of the most popular methods to study microglia. Both qualitative and quantitative assessments of microglial morphology are commonly used to make conclusions about neuroinflammation and microglial reactivity. Although a wide array of damage-associated microglial morphologies has been documented (Fig. 1), the exact functions of these distinct morphologies are not fully understood. Morphology is not the only indicator of microglial reactivity. Microglial reactivity is controlled by many coordinated factors; microglia express multiple cell surface receptors that regulate cell signaling and, in turn, microglial gene expression [8, 9]. Molecular techniques such as transcriptomics, ribonucleic acid (RNA) sequencing, immunohistochemistry, and flow cytometry are commonly used to examine microglial reactivity. In this review, we discuss how microglia morphology is not synonymous with microglia function, however, morphological outcome measures can be used to make inferences about function.

## Microglial morphologies

Microglial reactivity was long believed to mimic the M1 and M2 states of macrophages [10, 11], whereby M1 represents a pro-inflammatory phenotype and M2 an anti-inflammatory phenotype [12]. However, it is now understood that microglial reactivity occurs on a highly dynamic phenotypic continuum that involves the expression of many membrane-bound and secreted proteins [10, 13]. Initiation of microglial reactivity is a protective and reparative immune response. However, long-term persistence of glial reactivity and cytokine release causes a self-perpetuating state of chronic inflammation, exacerbates tissue damage, and can lead to neurodegeneration [14, 15].

Microglial morphology is highly plastic (Fig. 1) and changes depending on function and the signals present in the brain [4, 16]. There is heterogeneity of microglial morphology during pre- and postnatal development. In the mouse, microglia colonize neural tissue at embryonic days 7.5–8 and rapidly proliferate to reach a population of approximately 1200 by embryonic day 10.5 [17]. During early development, microglia are ameboid and undergo rapid cell division [18]. Flattened microglia are also observed in developing rodents along the walls of blood vessels [19]. In postnatal weeks 2–4, microglia in the mouse brain increase in size and fractal dimension, and assume a ramified morphology [20]. These characteristics and functions of microglia during postnatal development play a crucial role in monitoring and maintaining synapses and assist with the development and maintenance of neural circuits [21, 22].

In the adult mouse brain, microglia typically have a small cell body with highly ramified branches under noninflammatory conditions (Fig. 1A) [23–25]. Microglia actively protrude and retract their branches to detect environmental cues that signal damage to brain tissue [26, 27]. This ramified morphology allows single microglia to monitor large volumes of the parenchyma. Microglia are multifaceted in both function and morphology and are involved in many processes beyond surveillance. For example, microglia support neuronal circuit formation, synaptogenesis, and synaptic pruning [22]. Microglial signaling is also implicated in many disease processes including neurodegenerative, psychiatric, and neurodevelopmental disorders [22].

When microglia detect a pathological stimulus, they assume a reactive phenotype. Reactive microglia retract their branches and adopt a less complex structure with an enlarged cell body (Fig. 1B–E), a morphology associated with increased phagocytosis [4, 16, 28–30]. Microglia can adopt an ameboid morphology that has fully retracted branches and is associated with phagocytic activity [31]. In diseased states, microglia phagocytose: (1) extracellular proteins, including tau and amyloid beta; (2) extracellular organelles and cell fragments associated with cell death processes; (3) myelin; and (4) dead or dying cells that include other glial cells and neurons [32–36]. Ameboid microglia (Fig. 1C) are frequently observed in cases of severe or chronic inflammation [28, 37], or during development due to their role in phagocytosing neurons during neural network development [38]. Retraction of microglial branches increases the efficiency of migration to the site of damage, and increases phagocytic abilities [39].

Microglial reactivity is not a binary occurrence. There are intermediate microglial morphologies that fall between the two extremes of ramified and ameboid. For example, bushy microglia have a large cell body surrounded by short, stubby branches [39]. Perhaps similar, but more complex in appearance than a bushy morphology, hyper-ramified microglia have been observed in both injured and noninjured animal models. Recent studies linking microglia morphology with function have shown hyper-ramification of microglia is more common in mice with a stress resilience phenotype [40]. Hyper-ramified microglia have also been associated with other behavioral phenotypes. There is an increased abundance of hyper-ramified microglia in rats selectively bred to be more passive compared to rats bred to be highly exploratory [41]. After a diffuse traumatic brain injury in both rats and pigs, hyper-ramified microglia have been observed remote to the injury site [4, 42]. Another intermediate microglial morphology associated with pro-inflammatory conditions is hyper-ramified microglia, which have been associated with dendritic spine loss [43]. Another pathology-associated microglial morphology is rod microglia (Fig. 1D), which have not been associated with a specific function to date [44–46]. In severe cases of inflammation, microglia can coagulate and contribute to glial scar formation (Fig. 1E). This collection of reactive glia around focal points of tissue damage is seen following traumatic brain injury, stroke, and neurodegeneration [47].

Dystrophic microglia are another distinct morphological phenotype associated with neurodegenerative diseases and aging. Dystrophic microglia have a fragmented appearance and increase in abundance in patients with neurodegenerative diseases. It has been proposed that dystrophic microglia have undergone adaptations that promote a loss of function and represent a senescent phenotype [48]. Dystrophic microglia, positive for senescence markers, have been reported after traumatic brain injury [49]. Impaired microglial phagocytic activity and increased neuroinflammation also occur after brain injury [49]. Therefore, dystrophic microglia are likely involved in an important pathological paradigm between brain injury and increased risk of neurodegeneration.

## Endogenous factors that affect microglial morphology

Microglial morphology is affected by sex as shown by numerous preclinical studies, many of which have contrasting results. One translational study showed more phagocytic microglia at postnatal days 2–3 in female rats compared to males, however, found no differences in microglial morphology between sexes [50]. Numerous studies have reported that male rodents have an increased number of ameboid, and reactive microglia compared to females in early development [51, 52]. There are also more reactive microglia in the preoptic area in male rodents during postnatal development, compared to females, which may be linked to the formation of neuronal circuits and male sexual behaviors [51, 53], reviewed [54]. However, it has been shown that during later development (postnatal days 30–60), female rats have more reactive microglia than males, highlighting that microglia are regulated by both sex-specific and developmental processes [52]. For an in-depth review of this topic, please see [55].

Sex differences in microglial morphology have also been observed in the context of disease models. After neonatal ischemia, male mice have more ameboid microglia than females suggesting a more robust inflammatory response [56]. After traumatic brain injury in adult mice, female shams have a greater number of microglia than male shams, but there is no support for sex differences in microglial morphology [57]. In contrast, it has been shown that lipopolysaccharide administration drives changes in microglial morphology in male mice but not females [58]. These contrasting results suggest that the type of inflammatory stimulus, and the developmental time at which the stimulus is administered, may cause different responses in males and females.

The circadian rhythm and sleep are endogenous regulators of microglial morphology and are biological variables in the experimental design of morphology studies. During the inactive period, cortical microglia have a ramified morphology with longer and more complex branches than during wake [59]. The circadian fluctuations in microglial morphology are thought to be dependent on the cyclic expression of microglia-specific P2Y12, and blocking P2Y12 with a blood–brain barrier permeable inhibitor has been shown to stunt circadian fluctuations in microglial morphology [59, 60]. Circadian regulation of microglial morphology is maintained during constant darkness and does not rely on entrainment (i.e. diurnal light cue), which supports the hypothesis that time-of-day changes in microglial morphology may be driven by functions that are dependent on intrinsic cellular circadian rhythms [60, 61]. Daily fluctuations in microglial morphology are also dependent on clock gene expression, further supporting that microglia morphology and function are impacted by circadian rhythms [61]. Region-specific morphological observations have been made in the hippocampus [62] and the somatosensory cortex [63] following a secondary inflammatory stimulus. For an in-depth review of microglial circadian biology see [61].

## Methods to identify states of microglial reactivity

There are many quantitative and qualitative techniques that are used to examine microglia morphology. Qualitative techniques commonly involve assigning individual microglia cells into categories based on their morphology (e.g. ‘reactive’, ‘ameboid’, and ‘resting’) by eye. Qualitative microglia morphology techniques often involve bias and inconsistent scoring due to the continuous nature of microglial morphology. Quantitative techniques to examine microglia morphology from immunohistochemistry experiments involve quantifying the hallmark features of microglial morphology. The stain intensity/amount of staining is frequently quantified to represent the change of expression of markers of microglial reactivity. However, quantification of staining alone does not permit morphological conclusions. More in-depth quantitative morphological studies often examine cell body size, ramification/branching, and complexity of the branching pattern. Applying a technique that is appropriate for the study design and hypothesis is important when trying to avoid erroneous conclusions [64]. Machine learning techniques have also been developed for rapid classification of predefined microglial morphologies [65].

Microglial morphology, both *in vitro* and *in vivo*, is commonly assessed using immunocytochemistry or immunohistochemistry, respectively. Staining for microglial markers allows for spatial visualization of the proteins that the microglia are expressing. Commonly, ionized calcium-binding adapter molecule 1 (Iba1) is used to label microglia [4, 66]. There is some debate about whether Iba1 is expressed by all microglia [67]. Reactive microglia have higher levels of Iba1 expression [66, 68]. Therefore, quantifying the amount of Iba1, using either genetic or immunohistochemical approaches, is a popular measure of microglial reactivity. Importantly, the presence of Iba1 alone is not a marker of microglial reactivity. One of the limitations of using Iba1 is that the Iba1 antibody does not exclusively bind to microglia. Iba1 is expressed by microglia and macrophages, in both ramified and reactive states [24]. Depending on the context in which Iba1 is used, this may or may not limit the conclusions of a study. For example, if the study is purely examining whether there was an increase in inflammation, the infiltration of macrophages may not confound the conclusion.

Other commonly used surface markers, expressed predominantly by microglia, are the fractalkine receptor CX3C-chemokine receptor 1 (CX3CR1) and colony-stimulating factor 1 receptor (CSF-1R) [69–72]. CX3CR1 has been fluorescently tagged to enable microglia visualization [26, 27, 73, 74]. CSF-1R is essential to the survival of microglial cells and is often considered to be exclusively expressed by microglia, however, it is also expressed by myeloid cells, including subsets of peripheral macrophages [71, 75, 76]. Not only are microglia-specific markers useful to separate microglia from the general cell population, but these markers have also been used as targets to deplete microglia *in vivo*. CSF-1R has been pharmaceutically, and genetically, targeted to deplete microglia [76–79]. However, depleting microglia using PLX5622, an orally bioavailable CSF-1R inhibitor added to rodent chow, showed that CSF-1R inhibition also results in the depletion of subset of peripheral CD115 + monocytes [76].

For studies that require histological discrimination between microglia and infiltrating macrophages, transmembrane protein 119 (TMEM119) is a useful marker. TMEM119, which is specifically expressed in microglia, combined with Iba1 allows discrimination between Iba1 + microglia and macrophages due to the lack of TMEM119 expression in macrophages [24]. The function of TMEM119 after injury to the brain is currently unknown [80, 81]. In humans, TMEM119 has been shown to be expressed in specific subsets of microglia, so in clinical studies, this marker may have limited utility as a general marker for microglia in morphology-based studies [81]. One postmortem Alzheimer’s disease study found that TMEM119 was exclusively expressed on microglia that were Iba1^+^ and cluster of differentiation (CD)68^+^ which had both ramified and ameboid morphologies [81]. However, another study found that TMEM119 was not a reliable marker of microglia and cautioned TMEM119 use in microglia function and morphology studies [82]. Contrary to these clinical studies, TMEM119 is widely expressed by all microglia in mice [83], suggesting TMEM119 labeling of microglia may be species-specific, or poor antibody affinity in human tissue could be a potential confound. It is possible that the utility of TMEM119 as a microglial marker differs between injury types; in mice, TMEM119 is increased in microglia after optic nerve crush and lipopolysaccharide injection, but *tmem119* transcripts decrease in microglia after intracerebral hemorrhage [82, 84]. Similarly, TMEM119 decreases in reactive microglia after traumatic brain injury when examined with western blot and immunohistochemistry, but *tmem119* gene expression increases [85].

Other markers can be used in combination with detailed Iba1 morphology to indicate the inflammatory status and may facilitate the association of morphology with function. CD68 is a marker for macrophages, including microglia [86]. Microglia upregulate their expression of CD68 during reactivity [86]. Therefore, increased levels of CD68, localized to microglia, suggest more microglial reactivity. Similarly, CD11b is expressed by microglia and is upregulated during microglial reactivity [87]. Another histological marker of microglial reactivity is complement component 1q (C1q). Microglia are the main producers of C1q throughout the CNS. Although C1q exists at a low level in highly ramified microglia, C1q is rapidly upregulated after microglia detect a pathological stimulus and become reactive [88]. Under physiological conditions, microglia do not express major histocompatibility complex II (MHC II), however, microglia upregulate the expression of MHC II under inflammatory conditions [89]. OX6 is an MHC II marker that has been used to identify reactive, and rod-like microglia [90]. OX6 expression is also present in macrophages [91, 92]. MCH II expressing (OX6+) cells were shown to have an ameboid morphology in the brain and retina [91, 93]. Another marker used to detect microglial reactivity is HLA-DR. HLA-DR is a molecule of the major histocompatibility complex, an antigen presentation mechanism that displays foreign proteins to CD4^+^ T cells. It has been shown that microglia upregulate their expression of HLA-DR after inflammatory stimulation [94, 95].

When reactive, microglia downregulate some homeostatic markers, the loss of which can be correlated with function. P2RY12 is highly expressed in ramified microglia and decreases as microglia transition to a reactive phenotype (e.g. after brain injury, aging, multiple sclerosis, and Alzheimer’s disease) [96–98]. Therefore, P2RY12 could be used to detect a reduction in inflammation, in combination with other markers. A comprehensive list of microglial markers is beyond the scope of this morphology-focused review. For an in-depth review of microglial surface markers associated with specific injury types, see review [69].

Aside from *ex vivo* immunohistochemistry-based experiments, *in vivo* techniques can be used to examine the changing morphology of microglia in real time. Multiphoton microscopy can be performed on fluorescently tagged microglia. This permits acquisition of sequential images, allowing examination of the temporal microglial morphological response. Microglia can be fluorescently tagged via multiple mechanisms, including genetic knock-in [26, 27, 74, 99, 100], viral, cre-reporter line [101], and calcium indicators [102]. The development of live-imaging techniques to examine microglia has greatly increased the understanding of microglial morphology. It has been shown that ramified microglia rapidly extend and retract their processes at a rate of 2.2 ± 0.2 µm per minute [26, 27, 103]. Classic *ex vivo* immunohistochemical methods detected that microglia have multiple morphological appearances, however, transitions between these morphologies cannot be visualized in postmortem tissue. The benefits of an immunohistochemical approach are that multiple markers can be used which may provide more detail about the functional state of the microglia. *In vivo* imaging of fluorescently tagged microglia also involves a cranial window surgery which can induce an inflammatory response [27, 99, 103]. The maximal penetration depth of a multiphoton microscope is <1 mm which allows good spatial visualization of microglial cells and processes, however, the imaging depth is currently limited to between 500 µm and 1 mm [103]. Further, under anesthesia, microglial branches can elongate which makes the microglial response appear diminished [104–106], or anesthetics can induce a reactive phenotype with shortened branches [107]. However, to overcome this confound, some studies have image microglia in the absence of anesthesia [104]. Another group successfully used two-photon to image microglia through a thinned section of skull in the absence of a craniotomy [27]. The thinning of the skull reduces the amount of surgically induced inflammation. Together, all these experimental factors influence the morphological data acquired from two-photon photomicrographs. More recently, ‘miniature microscopes’ have been developed which allow examination of genetically tagged cells in the freely moving, awake mouse [108, 109]. The use of miniature microscopes also requires a cranial window surgery, and the microscope (approximately 2.5 g) is mounted to the head of the animal [109]. The use of head-mounted microscopes combats the issues of the administration of anesthesia during classic two-photon imaging which can alter microglial morphology.

## Methods to measure microglial morphology

There are many contrasting methods to analyze microglial morphology and differences in these methods can drive divergent scientific conclusions [64]. Often, microglial reactivity is measured through immunohistochemistry followed by calculating the amount of staining as a percentage or intensity [64]. The amount of staining can be sensitive to markers that are upregulated or downregulated when microglia are reactive (Iba1, TMEM119, CD68, CD11b, C1q, OX6, HLA-DR, and P2YR1), providing some information on the functional state of the microglial cell. However, calculating the amount of staining present can be affected by factors such as staining quality and regional changes in microglia expression (e.g. white matter tracts). For these reasons, comparisons across studies and laboratories are cautioned, and immunohistochemical and imaging procedures must be carefully considered when making scientific conclusions [64].

Other methods have been used to investigate the ramification and cell body size of microglial cells. Methods such as skeletal analysis allow assessment of the hallmark features of microglial reactivity, such as shortening of microglial branches/less ramification and enlarged cell soma size. There are many different programs, such as image segmentation to isolate microglial cells and facilitate microglial tracing, that allow measurement of microglial branches and cell body size [4, 110–112]. Measures of microglial morphology, especially when used in combination with functional markers (e.g. CD68 and C1q) can provide a comprehensive overview of the reactivity status of microglia. Additionally, other methods using fractal dimension have been created to analyze the pattern complexity of the microglia’s shape and branching pattern (reviewed [64, 111]). More recently, there have been extensive efforts to automate microglial morphology analysis. One study used a high throughput method called morphOMICS which offers a screening method with multiple outcome measures to understand the morphology of microglial populations on a larger scale [110]. Results using morphOMICS indicated that the morphology of microglia was brain region and sex specific, as discussed above [110]. For a comprehensive review of microglial morphology methods, please refer to [113].

In summary, there is no perfect method to examine microglial morphology. Therefore, study designs that include a microglial morphology outcome measure should be tailored to answer specific questions and a combination of markers and imaging techniques should be considered. With the wealth of available analysis techniques, it should be cautioned that appropriate data handling, outcome measures, and statistical approaches should be used to obtain accurate biological results.

## Microglial reactivity beyond morphology

The term ‘microglial reactivity’ is not interchangeable with ‘reactive microglial morphology’. There are many other features of microglia that indicate reactivity and not morphology. Here, we will briefly introduce some methods that can be used to examine markers of microglial reactivity; however, a comprehensive list of nonmorphological techniques is beyond the scope of this review. For example, single-cell RNA sequencing has been used to identify microglial signatures that show distinct microglial populations within the brain under inflammatory and noninflammatory conditions [114]. The identification of diverse subpopulations that change during development, aging, and after injury conflicts the binary classification of microglia into M1-like and M2-like phenotypes [114]. RNAScope and fluorescent *in situ* hybridization techniques are also used to visualize pro- and anti-inflammatory markers, including cytokines, on microglial cells [115]. Identification of pro- and anti-inflammatory markers on microglia increases the understanding of microglial responses to tested stimuli. Similarly, flow cytometry on dissociated brain tissue can be performed to identify inflammatory markers specific to microglia [76]. Using a combination of markers, flow cytometry can discriminate microglia (CD11b^+^ and CD45^med^) from monocyte-derived macrophages (CD11b^+^ and CD45^high^) [116]. However, the morphology of microglia cannot currently be examined using flow cytometry.

Clinically, it is difficult to examine cellular morphology during life. Neuroimaging techniques are used to examine inflammation and glial reactivity in living tissue. Positron emission tomography (PET) is an *in vivo* imaging technique that can be used both preclinically and clinically [117]. Specifically, activation of the immune response can be examined by looking at translocator protein (TSPO) which is expressed by reactive microglia [118]. The radioligand PK 11195 binds to TSPO enabling its detection with PET [118, 119]. Magnetic resonance imaging (MRI) is another neuroimaging technique that has been used to examine microglial activity in living brain tissue. A recent study used microglial and astrocytic fingerprints, unique signal combinations created for tissues of interest [120], to examine glia in living rats [121]. The use of branch density measurements showed changes in microglial reactivity after a hippocampal lipopolysaccharide injection [121]. Garcia-Hernandez *et al*. also demonstrated this technique could be adapted for use in humans.

## Conclusions

Microglial morphology is an easy-to-apply, consistent measure to examine microglia under experimental conditions. However, quantification of microglial morphology only scratches the surface of the biochemistry underlying the microglial response to insult. Therefore, the results from morphological studies should not be overinterpreted. For a comprehensive examination of the reactive status of a microglial cell, both histological and genetic approaches should be combined. However, the importance of quality immunohistochemistry-based analyses should not be overlooked as they can succinctly answer many research questions.
